# Silkworm Enzyme Hydrolysates Improve Memory in MCI Models via CREB-BDNF Signaling and Enhanced Brain Mitochondrial Function

**DOI:** 10.3390/nu17122044

**Published:** 2025-06-19

**Authors:** Yoo-Hee Kim, Nguyen Phuong, Nguyen Minh Anh Hoang, Hye-Jin Kim, Moo-Yeol Baik, Young Ho Koh

**Affiliations:** 1 Ilsong Institute of Life Science, Hallym University, Seoul 07247, Republic of Korea; yooii@hallym.ac.kr (Y.-H.K.); phuongnguyenvspt@gmail.com (N.P.); 2 Department of Biomedical Gerontology, Hallym University, Chuncheon 24252, Republic of Korea; minhanhhoang20598@gmail.com; 3 R&D Group, Maeil Health Nutrition Co., Ltd., Pyeongtaek 17714, Republic of Korea; hyejink@maeil.com; 4 Department of Food Science and Biotechnology, Kyung Hee University, Yongin 17104, Republic of Korea; mooyeol@khu.ac.kr

**Keywords:** HongJam, enzyme hydrolysates, mitochondria, programmed cell death, pyroptosis

## Abstract

**Background/Objectives**: This study investigated whether enzymatic hydrolysis enhances the cognitive benefits of HongJam (steamed mature silkworms) and explored the underlying mechanisms. A marker compound of enzyme-treated HongJam was also identified to support quality control. **Methods and Results**: Mice were supplemented with Golden Silk HongJam (GS) or its enzyme hydrolysates (GS-EHS). Behavioral tests showed both improved fear-aggravated memory, with GS-EHS producing similar or greater effects at lower doses. GS-EHS activated the cyclic AMP response element binding protein/brain-derived neurotrophic factor signaling pathway and mitigated scopolamine-induced mitochondrial dysfunction by enhancing mitochondrial complex activity and ATP production. It also increased esterase activity, reduced reactive oxygen species, and modulated programmed cell death by suppressing apoptosis while promoting autophagy and unfolded protein response pathways. These changes led to reduced endoplasmic reticulum stress and neuroinflammation. Mass spectrometry identified glycine-tyrosine dipeptide as a potential bioactive marker. **Conclusions**: GS-EHS enhances cognitive function by improving mitochondrial activity, reducing oxidative stress, and regulating programmed cell death. Enzymatic hydrolysis appears to increase the bioavailability of active compounds, making GS-EHS effective at lower doses. The glycine–tyrosine dipeptide may serve as a marker compound for standardizing GS-EHS based on its cognitive-enhancing properties.

## 1. Introduction

The rapid progression of biomedical science and technology has significantly increased human life expectancy in developed countries [1]. The increase in human life expectancy has inevitably led to an increase in the elderly population and the number of people with incurable diseases such as dementia [2]. Mild cognitive impairment (MCI) is a transitional stage between normal aging and dementia, characterized by noticeable declines in cognitive abilities—particularly memory, attention, and concentration—while daily functioning remains largely preserved. The progression rate to Alzheimer’s disease (AD) in normal elderly population is around 1 to 2%, but 10 to 15% of mild cognitive impairment (MCI) patients progress to AD every year [3]. Therefore, preventing cognitive decline in MCI patients is the best way to prevent their progression to AD [4]. Emerging evidence suggests that functional foods rich in bioactive compounds may support cognitive health by improving neuronal function, reducing oxidative stress, and modulating neuroinflammation, thereby potentially slowing the progression from MCI to AD [5].

HongJam is a health-promoting food made by steaming mature silkworms to make them edible for human consumption, and it is currently marketed as a natural health product in the Republic of Korea [5]. Its nutritional composition includes approximately 70% proteins, 15% lipids, 3% minerals, and 3% phytochemicals, including vitamins [6]. The silk glands of mature silkworms, which occupy a large portion of the abdomen, are filled with silk proteins, and are a major source of protein in HongJam. Among these silk fiber proteins, fibroin heavy chain (5263 amino acids), fibroin light chain (262 amino acids), and p23 protein form a stable macro-molecular complex with a molecular weight of approximately 2.3 MDa, in a molar ratio of 6:6:1 [7].

Previous studies have shown that enzymatic hydrolysate of silk proteins, typically extracted from silkworm cocoons, can improve memory performance in both animal studies and human clinical trials [7,8,9]. Enzymatic hydrolysis breaks down large, complex proteins into smaller peptides and amino acids, thereby increasing their solubility, absorption, and bioavailability in the body. These smaller peptides are more easily transported across biological membranes and may exhibit enhanced biological activity, including neuroprotective effects.

We have previously reported that HongJam had a memory-improving effect in an MCI animal model by enhancing mitochondria functions [10,11] and lowering blood cholesterols [12]. However, no preclinical study has investigated whether enzymatically hydrolyzed HongJam, prepared using edible enzymes, retains or enhances these memory-improving effects. In this study, we tested the hypothesis that enzymatic hydrolysis of HongJam would enhance its cognitive benefits in MCI models through mechanisms involving improved mitochondrial function and neuroprotection. To this end, we examined the cognitive-enhancing effects of HongJam hydrolysate in an MCI mouse model and explored the underlying molecular and cellular mechanisms.

## 2. Materials and Methods

### 2.1. Purchase Golden Silk HongJam and Production of Its Enzymatic Hydrolysate

Freeze-dried Golden Silk-HongJam powders (GS), made from Golden Silk mature silkworms that produce yellow cocoons, were purchased from TaeYang Farm (Yeongdeok-gun, Gyeongsangbuk-do, Republic of Korea). The GS-enzymatic hydrolysate (GS-EHS) was provided by Maeil Health Nutrition (Seoul, Republic of Korea). To produce GS-EHS, GS was dissolved in distilled water and extracted by boiling at 85–95 °C for 60 min. The extract was adjusted to become pH 7.0–7.5 using NaOH. Alcalase 2.4 L (Novozyme, Bagsværd, Denmark) was added into the solution at a concentration of 120 μL per gram of raw material (enzyme-to-substrate ratio of 120 μL/g, *v*/*w*), and enzyme hydrolysis was conducted at 45–55 °C. The reaction was terminated by heating the solution to 85–95 °C. Following hydrolysis, the mixture was filtered through filter paper to remove unhydrolyzed residues, sterilized by heat, and dried using a spray dryer.

According to the Korean Food and Drug Administration (KFDA), silkworms are classified as food and are not subject to daily intake limits for general consumption. However, when used as a health functional food, the KFDA allows a maximum daily intake of 2.7 g of freeze-dried silkworm for a 60 kg healthy adult. Additionally, processed foods made using approved food ingredients or food additives—such as GS and the enzyme Alcalase 2.4 L—are exempt from toxicity testing [5]. The dosage of GS-EHS used in this study, when converted to a human equivalent, is within the KFDA’s permitted intake range.

### 2.2. Analysis Protocol for the Marker Compound of GS-EHS

GS-EHS was analyzed using ultra-high performance liquid chromatography-tandem mass spectrometry (UHPLC-MS/MS) (Vanquish UHPLC and Q Exactive Plus LC-MS/MS, Thermo Fisher Sci., Waltham, MA, USA), equipped with an AdvanceBio peptide mapping column (2.1 × 100 mm, 2.7 µm, Agilent Technologies, Santa Clara, CA, USA). The analysis was based on peptide profiling and sequencing methodologies. Quantitative LC-MS and LC-MS/MS analyses were performed by Proteinworks (Daejeon, Republic of Korea, http://www.proteinworks.co.kr, accessed on 21 May 2025).

Peptide sequencing of the glycine–tyrosine (GY) dipeptide was confirmed using LC-MS/MS analysis through de novo sequencing. A calibration curve for quantification was constructed using five concentration points ranging from 0.0 to 2100 ng/mL. The glycine–tyrosine (GY) dipeptide was identified as the marker compound and its concentration was found to be approximately 0.1% (*w*/*w*) of the total GS-EHS content (see Figure S1), supporting its use as a standardized marker compound for quality control. To ensure analytical reliability, three independent lots of GS-EHS were analyzed to validate the method’s specificity, linearity, accuracy, and precision, following the Association of Official Analytical Chemists guidelines. Consistent marker content across the batches confirmed the robustness of the quantification method.

### 2.3. Animals and Experimental Substances

**Figure 1:**
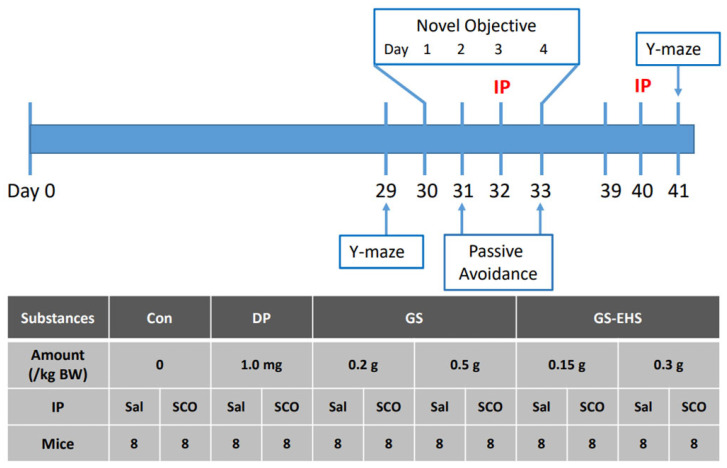
T Experimental design for behavioral analysis of SCO-IP-induced MCI animal models and the oral-administered amounts of substances used in this study. Each mouse was numbered from 1 to 96. Using the lottery method, 16 mice per group were alternately selected and assigned to either the Sal-IP or SCO-IP group. For the double-blinded tests, an independent individual administered various feeds and performed IPs to the mice without knowledge of their content. Additionally, the mouse cage position and the order of IPs were changed daily to avoid potential confounding effects. After supplementing various test substances for 28 days, behavioral experiments were conducted. On the 29th and 41st days, a Y-maze test was performed to assess changes in spatial memory induced by Sal- or SCO-IP. From the 30th to the 33rd day, a novel object recognition assay was conducted. The passive avoidance test began on the 31st day and concluded on the 33rd day. The Sal- or SCO-IP were administered twice, on the 32nd and 40th days. Each group consisted of 16 animals, and the experiments were conducted by randomly assigning them to either the Sal- or SCO-IP treatment. Con: the control subgroup, DP: donepezil subgroup, GS: GS subgroup, GS-EHS: GS enzyme hydrolysate group, MCI: mild cognitive impairment, Sal-IP, intraperitoneal injection of saline, and SCO-IP: intraperitoneal injection of scopolamine.

The experimental Animal Ethics Committee at Hallym University (HMC-2022-0-0325-07) gave permissions and guidance for conducting this study. Four-week-old male C57BL/6J mice provided from Saeron Bio. (Uiwang-si, Republic of Korea) were habituated in animal facilities for one week. The amounts of oral-feeding supplements were decided based on previous publications [10,11]. The positive control drug, 1.0 mg/kg body weight (BW) of donepezil (DP, Merck KGaA, Darmstadt, Germany), 0.2 and 0.5 g/kg BW of GS, and 0.15 g and 0.3 g/kg BW of GS-EHS provided from Maeil Health Nutrition were orally administrated. The planning, conducting, and analyzing of data for animal experiments followed the guidelines from ADDRESS 2.0 [13]. The control group (Con) was only administered a 0.025% MC solution. Ninety-six C57BL/6J mice were randomly allocated to each group as depicted in Figure 1. Each group consisted of 16 mice, randomly divided into two subgroups: intraperitoneal injection of saline (Sal-IP) or scopolamine (0.75 mg/kg BW, Tokyo Chemical Industry, Tokyo, Japan) (Sco-IP). To minimize observer bias, both the individual conducting behavioral tests and the data analyst were blinded to group allocations. Mice were housed in cages with four animals per cage. They were orally fed daily and received two IPs without special measures, as these procedures caused only momentary pain. None of the mice exhibited sudden weight loss exceeding 20% or abnormal behaviors.

### 2.4. Behavior Test Protocols

Previously published animal behavior assay protocols were used [10,11]. Designs for the animal behavior analysis in MCI animal models are depicted in Figure 1.

#### 2.4.1. Y-Maze Spontaneous Alteration Assays

After 28 days of oral administrations of test substances, Y-maze assays were performed twice on Days 29 and 41 as previously published [11]. The Y-maze apparatus (Jeungdo Bio & Plant Co., Ltd., Seoul, Republic of Korea) comprises three arms connected at the center at equal angle. A mouse was gently placed in the center of the Y-maze, and its movements were recorded for 10 min. When a mouse entered two different arms consecutively without returning to the previous arm, it was counted as a spontaneous alternation. The equation for calculating spontaneous alteration rate (SAR) is below.

SAR = a number of spontaneous alteration/(total numbers of arm entries − 2) × 100

#### 2.4.2. Passive Avoidance Tests (PATs)

After 30 days of oral administration of test substances, PATs were conducted as previously published [10,11]. Thirty minutes before starting the PATs, mice were transferred to a behavior analysis room. On the first day, the mice were placed in a light compartment of a PAT device (Jeungdo Bio & Plant Co., Ltd.) and allowed to explore freely for two minutes. The next day, 30 min before starting the training session, mice were administered either Sal- or SCO-IP (0.75 mg/kg body weight). The mouse was placed in the light compartment for 30 s, and then a door blocking the light and dark compartments of the PAT apparatus was opened. The time it took for the mouse to move completely, including its tail, into the dark compartment was measured. Once the mouse had entered the dark compartment, the door was closed and was given an electric shock of 0.4 mA for three seconds. On the final testing day, the mouse was placed in the light compartment for 30 s before opening the door. The time taken for the mouse to move from the light to the dark compartment was measured, or the trial was terminated if the mouse did not move within 300 s.

#### 2.4.3. Novel Objective Recognition Assays (NORs)

After 29 days of oral administration of test substances, NORs were performed as previously published [14]. For habituation during the 1st and 2nd days, mice were transferred to a behavior analysis room for 10 min near a NOR arena. On the 3rd training day, mice were transferred to the behavior analysis room 10 min before starting NOR tests. Two identical objects were placed in the NOR arena and mice were allowed to freely explore for 10 min. The final testing day, after 10 min waiting in the behavior analysis room, one of the old objects in the NOR arena was replaced with new objects and the mice were introduced and their behaviors were recorded for 10 min. The exploration times for each object were counted and preference ratios for each subject were calculated as follows:

The preference (%) = time to explore the individual object/total exploration time to objects × 100%,(1)

### 2.5. The Mouse Brain Dissections

Four days after completing the behavioral analysis, the mice were euthanized using a homemade CO_2_ euthanasia device, and perfusions were performed with ice-cold PBS for 30 min. The brains were then dissected and divided into seven parts: the cerebral cortex, the olfactory bulb, the frontal cortex, the hippocampus, the cerebellum, the striatum, and the brain stem, as previously described [10,11]. Obtained brain parts were used for the biochemical and molecular biological analyses described below.

### 2.6. Mitochondria Extraction, Mitochondria Complex I–IV Activity, and ATP Quantification Protocols

Previously published protocols were used [12,15]. Briefly, the cerebral cortex was homogenized with mitochondria extraction buffer [MEB; 0.01 mM HEPES; pH 7.2, 125 mM sucrose; 250 mM mannitol; 10 mM EGTA; 0.01% (*w*/*v*) BSA, Merck KGaA], and then centrifuged at 700× *g* for 10 min at 4 °C to collect the supernatant. To obtain mitochondria pellets, supernatants were further centrifuged at 10,000× *g* for 10 min at 4 °C. The pellets containing mitochondria were mixed with ice-cold MEB for measuring the activities of the mitochondria complex (MitoCom) I&II. For measuring the activities of MitoCom III&IV, ice-cold MEB containing 1 mM n-D-β-D maltoside (Merck KGaA) was used. Protein concentrations of mitochondrial samples were determined using the BCA assay (Thermo Fisher Sci.).

#### 2.6.1. MitoCom I&II Activity Assays

For the measurement of MitoCom I activity, MitoCom I assay buffer [25 mM phosphate buffer, pH 7.8, 0.35% bovine serum albumin (BSA), 60 μM dichlorophenolindophenol (DCIP), 70 μM decylubiquinone,1 μM antimycin A, 0.02 mM NADH, Merck KGaA] containing 5 mM NADH was mixed with 20 μg of the mitochondrial sample. After incubating at 37 °C for 4 min, the absorbance at 600 nm (A600nm) of mixed samples was measured using a Multiskan Go spectrophotometer (Thermo Fisher Sci).

For the measurement of MitoCom II activity, MitoCom II assay buffer (80 mM KH2PO4; pH 7.8, 0.1% BSA (*w*/*v*); 2.0 mM EDTA; 0.2 mM ATP; 80 mM DCIP; 50 mM decylubiquinone; 1.0 mM antimycin A; 3.0 mM rotenone, Merck KGaA) and 20 μg of the mitochondrial sample was mixed. After incubating at 37 °C for 10 min, 4.0 μL of 0.1 M KCN and 2 μL of 1.0 M succinate were added to the sample and then A600nm was measured for 5 min. The equation for calculating MitoCom I&II activity is as follows:

Activity = [ΔA600/min × V of assay (mL)]/[(ε of DCIP × V of sample (mL)) × (protein concentration)], ε of DCIP: 19.1 mM^−1^, (2)

#### 2.6.2. MitoCom III&IV Activity Assays

For the measurement of the MitoCom III activity, MitoCom III assay buffer (50 mM Tris-HCl pH 7.5, 0.1 mM decylubiquinone, 12.5 mM succinate, 2 mM potassium cyanide, 30 µM rotenone, 40 µM cytochrome C, and 4 mM NaN_3_) and 20 μg of the mitochondrial sample was mixed. The absorbance at 550 nm (A550nm) of the mixture was measured for 3 min at 30 °C. The MitoCom III activity was determined using the following equation:

Milli OD/min/µg protein = [(ΔA550nm of sample − ΔA550nm of blank)/min]/[V of sample (mL) × protein concentration],(3)

The activity of MitoCom IV was measured by adding a mixture of 0.22 mM ferrocytochrome cytochrome C (Merck KGaA) and 0.1 M dithiothreitol (DTT, Merck KGaA) reacted for 15 min at room temperature to the mixture of 5 μg of the mitochondrial sample and MitoCom IV assay buffer (10 mM Tris-HCl, pH 7.0, 120 mM KCl). A500nm of the mixtures was measured at 25 °C, with every 10 s for 7 cycles. The MitoCom IV activity was determined using the following equation:

Activity (µmol/min/mg) = (ΔA550nm × 0.2)/[(21.84 × 0.002) × (protein concentration)], 0.2: reaction volume (mL), 0.002: sample volume (mL), 21.84: ΔзmM between ferrocytochrome C and ferricytochrome C at 550 nm.(4)

#### 2.6.3. The ATP Quantification Protocol

The phenol-saturated TE buffer containing 15% chloroform and 10% dH_2_O was used to homogenize brain tissues, vortexed, and then centrifuged at 10,000× *g* for 5 min at 4 °C to collect the supernatant. The remaining precipitates were used for protein quantification. ATP assay buffer (50 μM luciferin,1.25 μg/mL luciferase, 1 mM DTT, 20.875 mM Tricine, 4.175 mM MHJO_4_, 0.835 mM EDTA, 0.835 NaN_3_, Merck KGaA), supernatants, and ATP standards were mixed. A Victor Nivo™ multimode plate reader (PerkinElmer, Waltham, MA, USA) was used to measure the luminescence of mixtures. A standard curve prepared using ATP standard solution was used to quantify for supernatants.

All biochemical results were normalized to the mean value of Con within the Sal-IP group.

### 2.7. Western Blot Analysis

Brain parts were homogenized with a protein extraction solution (Pro-PrepTM, iNtRON Biotechnology, INC. SeongNam, Republic of Korea) containing a Halt protease and phosphatase inhibitor cocktail (Thermo Fisher Sci.). After incubation on ice for 30 min, lysates were centrifuged at 16,000× *g* for 10 min at 4 °C to obtain supernatants. The protein concentrations of lysates were determined using the BCA methods (Bio-Rad Laboratories, Hercules, CA, USA). Equal amounts of protein lysates were separated by SDS-poly-acrylamide gel electrophoresis and then transferred to a nitrocellulose (NC) membrane. The membranes were probed with the following antibodies: mouse monoclonal anti-β-actin (1:5000, sc-47778, Santa Cruz Biotechnology, Inc., Dallas, TX, U.S.A.), rabbit polyclonal anti-brain-derived neurotrophic factor (BDNF, 1:1000, ab226843, Abcam, Cambridge, UK), mouse monoclonal anti-cyclic AMP response element binding protein 1 (CREB1, 1:250, sc-271, Santa Cruz Biotechnology, Inc.), and mouse monoclonal anti-phosphorylated-CREB1 (p-CREB1, 1:250, sc-81486, Santa Cruz Biotechnology, Inc.) antibodies. Digital images of membranes were acquired by using an iBright 1500 imaging system (Thermo Fisher Sci.) and processed using Adobe Photoshop 2022 (Adobe, San Jose, CA, USA). Intensities and areas of each band were calculated and then normalized with those of β-actin.

### 2.8. Total Esterase Activity Assay

Previously published total esterase activity assay was performed [11]. The tested mouse brains were homogenized with ice-cold 0.1 M phosphate buffer with 1.0% Triton X-100 (PBT) and centrifuged at 16,000× *g* for 10 min at 4 °C to harvest supernatants. No additional dilution of the supernatants was performed prior to the assay.

For the reaction, 20 μL of supernatants was mixed with 180 μL of assay buffer containing 0.5 mM 1-napthyl butyrate (NB), 0.01 M MgCl_2_, and 0.01% Nonidet P-40 (Merck KGaA) in 5.0 mM PB (pH 7.0). The protein concentrations in supernatants were measured by the BCA method and used for normalizing the activities which were measured at 320 nm for 10 min at 30 s intervals using a Multiskan GO (Thermo Fisher Sci.).

Total esterase activity (μmol/mil/min) = [ΔA320nm × Vreaction (mL) × dilution factors]/[ε × V sample (mL)]/sample protein concentration, ε for NB = 1.226544 mM^−1^ cm^−1^, (5)

### 2.9. Reactive Oxygen Species (ROS) Quantification Protocol

ROS amounts in the tested mouse brains were measured as previously published [12]. Supernatants were mixed with PBS and 10 μM 2’,7’-dichlorofluorescin diacetate substrate (Merck KGaA) was incubated for 30 min at 37 °C in the dark to prevent the photo-oxidation of the dye. A multimode plate reader (Victor NivoTM, Perkin Elmer, Waltham, MA, USA) was used to excite at 480 nm and detect the emission fluorescence intensities (FI) at 535 nm. ROS amounts were calculated by the following equation:

ROS amounts: FI/mg protein = (ΔFI × 0.2)/(0.05) × [sample protein concentration]),(6)

### 2.10. Real Time Quantitative PCR (RT-q-PCR) Protocol

Brain parts were homogenized in Tri-RNA reagent (FAVORGEN Biotech Corp., Ping Tung, Taiwan) and total RNAs were purified according to the manufacturer’s protocol. The quality and integrity of total RNAs were examined by measuring A260/A280 and 28S rRNA/18S rRNA ratios. After being treated with DNase I (Enzynomics, DaeJeon, Republic of Korea) to remove genomic DNA contaminants, the total RNAs were used for synthesizing cDNAs with Superscript IV (Thermo Fisher Sci.). Expression levels of 26 genes involved in apoptosis, unfolded protein response (UPR)/autophagy, synaptic plasticity, and inflammasome formation signaling were examined by using Go Taq-q-PCR master mix (Promega, Madison, WI, USA). The list of genes, nucleotide sequences of oligomers, and PCR conditions is provided in Supplementary Table S1. Three independent samples were used for each analysis. Gene expression levels were calculated using the 2^−ΔΔCT^ methods, with β-actin as a loading control.

### 2.11. Statistical Analysis

Statistical analyses were performed following Shapiro–Wilk normality tests to assess data distribution. Depending on the experimental design, one- or two-way repeated measured analyses of variance were used to evaluate statistically significant differences between groups. When significant main effects or interactions were detected, Tukey’s honestly significant difference test was used for post hoc multiple comparisons. All statistical analyses were conducted using Microsoft Excel (Microsoft, Redmond, WA, USA). Data are presented as the mean ± standard error of the mean (SEM). Different letters above the error bars in the figures indicate statistically significant differences at *p* < 0.05.

## 3. Results

### 3.1. Significantly Enhanced Fear-Aggravated Memory in the MCI Mouse Models Supplemented with GS-EHS

**Figure 2:**
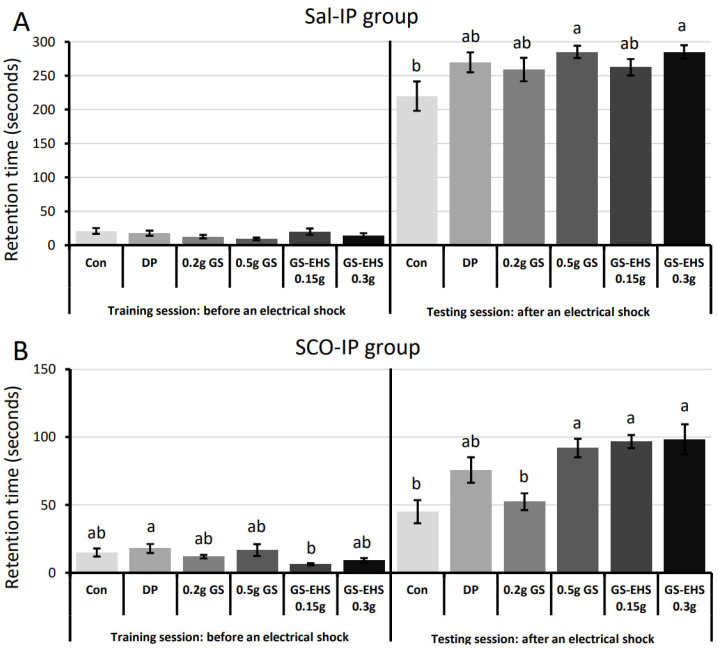
Enhanced fear-aggravated memory in MCI mice supplemented with GS and GS-EHS. (**A**). Sal-IP group: No significant effect of supplementation on retention time in the bright compartment during the training session (F_(5, 42)_ = 1.656, *p* = 0.166408). However, a significant effect was observed during the testing session (F_(5, 42)_ = 2.642, *p* = 0.03644). The retention time of the Con was significantly shorter than those of the 0.5 g GS and 0.3 g GS-EHS. (**B**). SCO-IP group: A significant effect of supplementation was observed during the training session (F_(5, 42)_ = 2.803, *p* = 0.028415). The retention time of the DP group was significantly longer than that of the 0.15 g GS-EHS group. A significant effect was also found during the testing session (F_(5, 42)_ = 8.284, *p* = 1.63 × 10^−5^). The retention time of the Con and 0.2 g GS in the SCO-IP group was significantly shorter than those of the 0.5 g GS 0.15 g GS-EHS, and 0.3 g GS-EHS. Different letters above the bars indicate significant differences (*p* < 0.05). Con: the control subgroup, DP: donepezil subgroup, GS: GS subgroup, GS-EHS: GS enzyme hydrolysate group, MCI: mild cognitive impairment, Sal-IP, intraperitoneal injection of saline, and SCO-IP: intraperitoneal injection of scopolamine.

The hippocampal learning and memory-enhancing effects of GS and GS-EHS using SCO-IP-induced MCI mouse models by performing passive avoidance tests (PATs), Y-maze, and novel object recognition (NOR) assays were investigated. No statistically significant difference in the latencies in the light chamber among in the Sal-IP group in the training session were observed (Figure 2A. F_(5, 42)_ = 1.656, *p* = 0.166). In the testing session, mice receiving 0.5 g GS and 0.3 g GS-EHS showed significantly longer latencies than the Con (Figure 2A. F_(5, 42)_ = 2.642, *p* = 0.03644).

In the SCO-IP MCI group, there were significant differences in latencies in the light chamber between the DP and the 0.15 g GS-EHS in the training session (Figure 2B. F_(5, 42)_ = 2.803, *p* = 0.028). However, the Con and the 0.2 g GS spent significantly less time in the light chamber than the 0.5 g GS, the 0.15 g GS-EHS, and the 0.3 g GS-EHS in the testing session. (Figure 2B. F_(5, 42)_ = 8.284, *p* = 1.6 × 10^−5^). These PAT results suggest that MCI mice supplemented with 0.5 g GS, 0.15 g GS-EHS, and 0.3 g GS-EHS had enhanced fear-aggravated memory compared to Con.

We conducted a study to compare changes in short-term spatial memory between the Sal- and SCO-IP groups using the Y-maze. A significant effect of the supplementation on spontaneous alternation rates (SPR) (*p* = 0.000825) was observed, but no effect of IP treatment (*p* = 0.749) or interaction (F_(5, 84)_ = 2.873, *p* = 0.019) in the Sal-IP group (Supplementary Figure S2A) was observed. In the SCO-IP group, the supplementation had a significant effect on SPR (*p* = 0.03), but not the SCO-IP (*p* = 0.055). No significant interaction effect was observed (Supplementary Figure S2B. F_(5, 84)_ = 1.101, *p* = 0.365).

Normal rodents show preferences to the new object, rather than the familiar object [14]. A novel objective recognition (NOR) assay was performed to investigate whether supplementation with GS or GS-EHS might change their preferences for novel objects (*p* = 1.08 × 10^−5^). No effect of IP treatment (*p* = 0.65) or interaction was observed. The supplementation significantly improved preferences for novel objects. There were significant effects of the supplementation on preference, but not by Sal- or SCO-IP (Supplementary Figure S3A. F_(5, 84)_ = 1.836, *p* = 0.114). No significant effects of the supplementation (*p* = 0.109), IP treatment (*p* = 0.373), or interaction (F_(5, 84)_ = 1.033, *p* = 0.404) were observed (Supplementary Figure S3B).

These learning and memory behavioral test results suggested that 0.5 g GS, 0.15 g GS-EHS, and 0.3 g GS-EHS enhanced hippocampal learning and memory.

### 3.2. Significantly Enhanced MitoCom I–IV Activities and ATP Amounts in MCI Mouse Brains Supplemented with GS-EHS

#### 3.2.1. MitoCom I–IV Activity Assay Results

**Figure 3:**
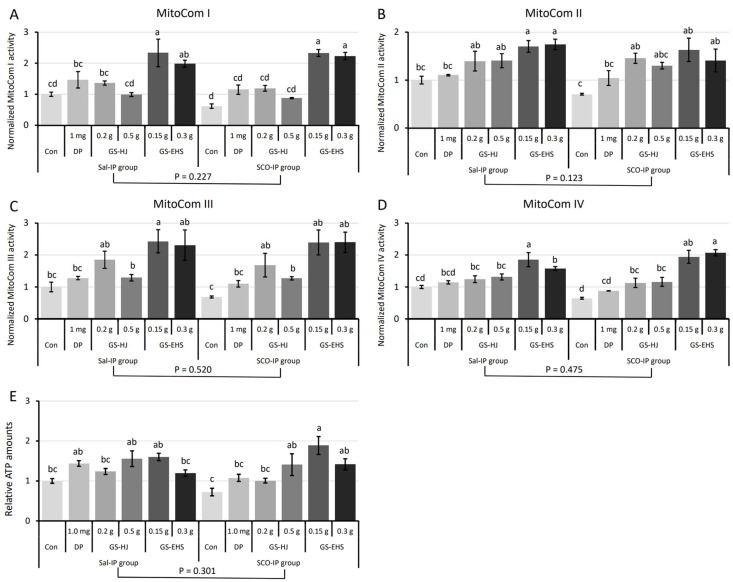
Reduced mitochondrial complex (MitoCom) I–IV activities and ATP levels in the brains of control (Con) mice under SCO-IP treatment were restored by GS-EHS supplementation. (**A**). MitoCom I: A significant effect of supplementation (*p* = 5.2 × 10^−9^) was observed, but no significant effect of IP treatment (*p* = 0.227) or interaction effect (F_(5, 24)_ = 0.882, *p* = 0.507). (**B**). MitoCom II: A significant effect of supplementation was observed (*p* = 6.2 × 10^−5^), but no significant effect of IP treatment (*p* = 0.123) or interaction effect. (**C**). MitoCom III: A significant effect of supplementation was observed (*p* = 9.7 × 10^−6^), with no significant effect of IP treatment (*p* = 0.51) or interaction effect (F_(5, 24)_ = 0.142, *p* = 0.98). (**D**). MitoCom IV: A significant effect of supplementation was observed (*p* = 4.2 × 10^−6^), with no significant effect of IP treatment (*p* = 0.476). However, a significant interaction effect was found (F_(5, 24)_ = 3.291, *p* = 0.021). (**E**). ATP levels: A significant effect of supplementation was observed (*p* = 4.6 × 10^−5^), with no significant effect of IP treatment (*p* = 0.300) or interaction effect (F_(5, 24)_ = 1.911, *p* = 0.13). Different letters above the bars indicate significant differences (*p* < 0.05). Con: control subgroup, DP: the donepezil subgroup, GS: GS subgroup, GS-EHS: GS enzyme hydrolysate group, MCI: mild cognitive impairment, Sal-IP, intraperitoneal injection of saline, and SCO-IP: intraperitoneal injection of scopolamine.

We investigated changes in mitochondrial activity in the brains of mice that consumed various types of HongJam substances. Significant effects of the supplementation were observed on mitochondrial complex (MitoCom) activities I–IV (MitoCom I: *p* = 5.2 × 10^−9^; MitoCom II: *p* = 6.17 × 10^−5^; MitoCom III: *p* = 9.68 × 10^−6^; MitoCom IV: *p* = 4.2 × 10^−9^). In contrast, neither Sal-IP nor SCO-IP treatments alone significantly affected these activities (all *p* > 0.1; Figure 3A–D). No significant interaction was found between the supplementation and IP treatment for MitoCom I–III activities (MitoCom I: F_(5, 24)_ = 0.883, *p* = 0.508; MitoCom II: F_(5, 24)_ = 0.536, *p* = 0.747; MitoCom III: F_(5, 24)_ = 0.142, *p* = 0.98). However, a significant interaction was observed for MitoCom IV (F_(5, 24)_ = 3.292, *p* = 0.02).

For MitoCom I, activity levels were significantly higher in the DP, 0.5 g GS, and 0.15 g GS-EHS in the Sal-IP group, as well as in the 0.15 g and 0.3 g GS-EHS in the SCO-IP group, compared to the Con in the SCO-IP group (Figure 3A). For MitoCom II and III, activity levels in the Con in the SCO-IP group were significantly lower than in multiple GS and GS-EHS in both Sal- and SCO-IP groups (Figure 3B,C). For MitoCom IV, GS and GS-EHS supplementation significantly increased activity compared to the Con in the SCO-IP in both groups (Figure 3D).

#### 3.2.2. ATP Quantification Assay Results

Brain ATP levels were also measured. Supplementation had a significant effect (*p* = 4.5 × 10^−5^), whereas IP treatment did not (*p* = 0.301), and no interaction effect was detected (F_(5, 24)_ = 1.912, *p* = 0.13). ATP levels in the Con in the SCO-IP group were significantly lower than in the DP, 0.5 g GS, and 0.15 g GS-EHS in the Sal-IP group, and 0.5 g GS, 0.15 g GS-EHS, and 0.3 g GS-EHS in the SCO-IP group (Figure 3E).

Overall, GS-EHS supplementation reversed SCO-IP-induced reductions in MitoCom I–IV activities and ATP levels and also enhanced these measures even under normal conditions (the Sal-IP group). These improvements in mitochondrial function suggest enhanced cellular energy metabolism and neuronal viability. Importantly, such bioenergetic restoration may contribute to the observed improvements in cognitive performance in GS-EHS-treated groups, as demonstrated in the PAT, Y-maze, and NOR behavioral assays. Enhanced mitochondrial activity and ATP production are closely linked to synaptic plasticity and memory formation, providing a potential mechanistic basis for the memory-enhancing effects of GS-EHS.

### 3.3. The Activated BDNF and CREB Signaling in the Mouse Brains Supplemented with GS-EHS

To investigate the mechanisms underlying the memory-enhancing effects of GS-EHS, we examined changes in the expression of key factors involved in memory-related signaling pathways in brain tissues.

#### 3.3.1. BDNF Quantification Results

**Figure 4:**
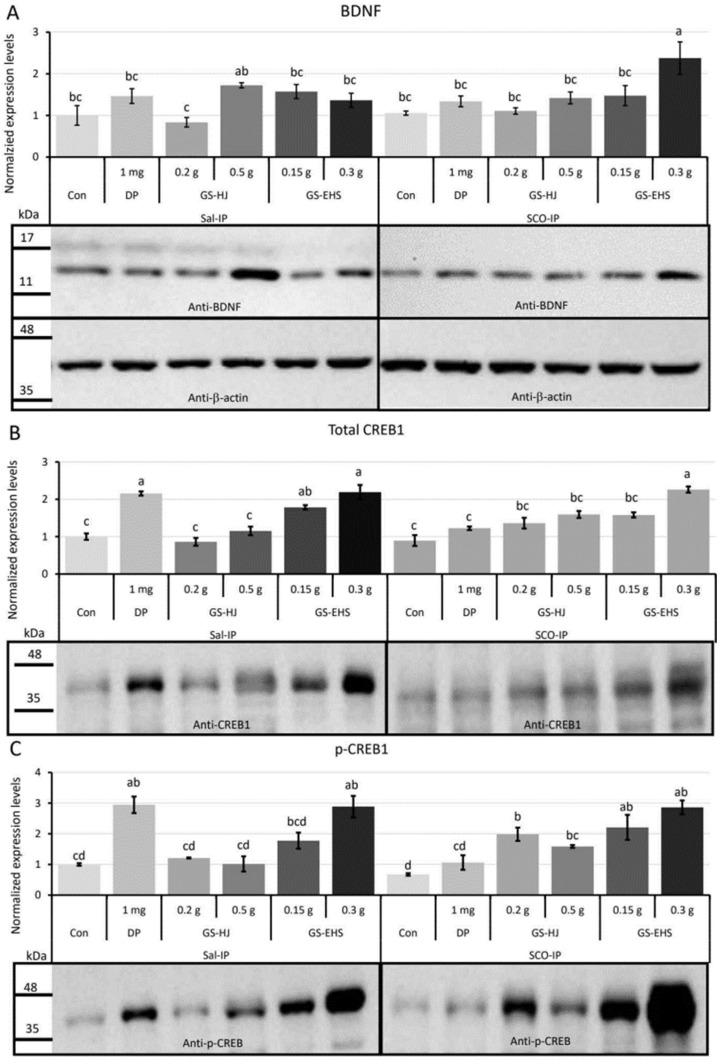
Expression levels of BDNF, total CREB1, and p-CREB1 were significantly increased in GS-EHS in both groups compared to the Con in the SCO-IP group. (**A**). BDNF: A significant effect of supplementation was observed (*p* = 0.002), with no significant effect of IP treatment (*p* = 0.811) and no interaction effect (F_(5, 24)_ = 1.718, *p* = 0.169). (**B**). Total CREB1: A significant effect of supplementation was observed (*p* = 1.7 × 10^−10^), with no significant effect of IP treatment (*p* = 0.543). A significant interaction effect was found (F_(5, 24)_ = 11.69, *p* = 8.4 × 10^−6^). (**C**). p-CREB1: A significant effect of supplementation was observed (*p* = 2.0 × 10^−7^), with no significant effect of IP treatment (*p* = 0.578). A significant interaction effect was found (F_(5, 24)_ = 8.708, *p* = 8.7 × 10^−5^). Different letters above the bars indicate significant differences (*p* < 0.05). BDNF: brain-derived neurotrophic factor, Con: the control subgroup, CREB1: cyclic AMP-response element binding protein 1, DP: donepezil subgroup, GS: GS subgroup, GS-EHS: GS enzyme hydrolysate group, p-CREB1; phosphorylated-CREB1, Sal-IP, intraperitoneal injection of saline, and SCO-IP: intraperitoneal injection of scopolamine.

A significant effect of supplementation was observed on the expression levels of BDNF, a neurotrophic factor known for its neuroprotective functions (*p* = 0.0019). However, there was no significant effect of IP treatment (*p* = 0.811) or interaction between supplementation and IP treatment (F_(5, 24)_ = 1.718, *p* = 0.169). The BDNF expression levels in the Con and 0.2 g GS within the Sal-IP group, and in the Con of the SCO-IP group, were significantly lower than in the 0.3 g GS-EHS under SCO-IP treatment (Figure 4A).

#### 3.3.2. CREB1 Quantification Results

CREB1, a transcriptional regulator involved in synaptic plasticity, learning, and memory, also showed significant changes in expression. Total CREB1 expression levels were significantly affected by supplementation (*p* = 1.68 × 10^−10^), with no significant effect of IP treatment (*p* = 0.543). However, a significant interaction effect was found (F_(5, 24)_ = 11.693, *p* = 8.37 × 10^−6^). The total CREB1 expression levels of the Con and 0.2 g GS in the Sal-IP group and the Con and DP in the SCO-IP group were significantly lower than those in the DP and 0.3 g GS-EHS in the Sal-IP group and the 0.3 g GS-EHS in the SCO-IP group (Figure 4B).

#### 3.3.3. p-CREB1 Quantification Results

p-CREB1 expression levels showed even greater differences. Supplementation had a significant effect (*p* = 2.0 × 10^−7^), whereas IP treatment had no significant effect (*p* = 0.579). A significant interaction effect was also observed (F_(5, 24)_ = 8.708, *p* = 8.1 × 10^−5^). The p-CREB expression levels in the Con, 0.2 g GS, and 0.5 g GS in the Sal-IP group and the Con and DP in the SCO-IP group were significantly lower than those in the DP and 0.3 g GS-EHS in the Sal-IP group and the 0.2 g GS, 0.15 g GS-EHS, and 0.3 g GS-EHS in the SCO-IP group (Figure 4C).

These results collectively suggest that GS-EHS supplementation activates the BDNF and CREB signal transduction pathways associated with neuroprotection and memory enhancement, which may contribute to its cognitive benefits.

### 3.4. Increased Total Esterase Activity and Reduced ROS Amounts in GS-EHS-Supplemented Mouse Brains

#### 3.4.1. Total Esterase Activity Assay Results

**Figure 5:**
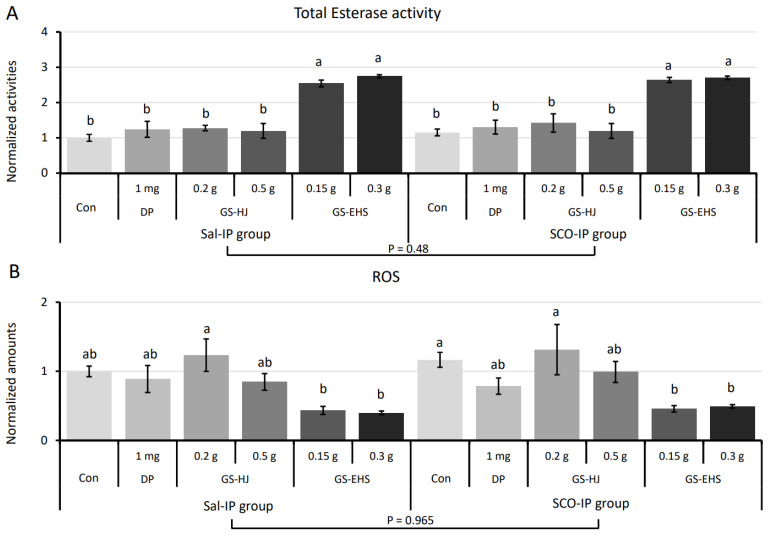
Enhanced total esterase activities and reduced ROS amounts in the brains of MCI mouse models supplemented with GS-EHS. (**A**). A significant effect of supplementation (*p* = 2.0 × 10^−17^) on total esterase activity levels in the brain, but no significant effect of IP treatment (*p* = 0.48) or interaction between supplementation and IP treatment (F_(5, 24)_ = 0.156, *p* = 0.977). (**B**). Significant effects of the substances (*p* = 4.6 × 10^−6^) and interaction (F_(5, 24)_ = 0.189, *p* = 0.965), but no significant effect of IP treatment (*p* = 0.464) on the amounts of ROS in the brains. Different letters above the bars indicate significant differences (*p* < 0.05). Con: the control subgroup, DP: donepezil subgroup, GS: GS subgroup, GS-EHS: GS enzyme hydrolysate group, MCI: mild cognitive impairment, ROS: reactive oxygen species, Sal-IP, intraperitoneal injection of saline, and SCO-IP: intraperitoneal injection of scopolamine.

Total esterases, including serine hydrolases, are abundant in the brain, and are critical for maintaining cellular and tissue homeostasis. Abnormal esterase activity is linked to various diseases, including dementia [16]. Supplementation with GS or GS-EHS significantly affected total esterase activity (*p* = 2.0 × 10^−17^), while IP treatment had no significant effect (*p* = 0.481), and no interaction effect was observed (Figure 5A, F_(5, 48)_ = 0.157, *p* = 0.977).

#### 3.4.2. Reactive Oxygen Species Quantification Results

Reactive oxygen species (ROS) are highly reactive metabolites that induce oxidative stress in cells [17]. GS/GS-EHS supplementation significantly reduced ROS levels (*p* = 4.6 × 10^−6^), with no significant effect of IP treatment (*p* = 0.463) or their interaction (F_(5, 48)_ = 0.189, *p* = 0.966). The 0.15 g and 0.3 g GS-EHS showed significantly lower ROS levels than the Con and 0.2 g GS groups in both Sal-IP and SCO-IP conditions (Figure 5B).

By enhancing total esterase activity and mitigating oxidative stress, GS-EHS supplementation may support cognitive function through improved cholinergic signaling and neuroprotection.

### 3.5. Supplementation GS-EHS Altered Expression Levels of Genes in Type I, Type II, and Inflammatory Programmed Cell Death Pathways in Mouse Brains

Expression levels of genes in type I (apoptosis), type II (autophagy/UPR), and inflammatory (pyroptosis) programmed cell death (PCD) pathways listed in Table S1 [18] in brains of the Sal-IP and SCO-IP groups were analyzed by RT-q-PCR.

#### 3.5.1. Type I PCD-Related Gene Expression Changes (Bax, Bak1, Casp9, TNF-α, Fas-L, PARP1)

**Figure 6:**
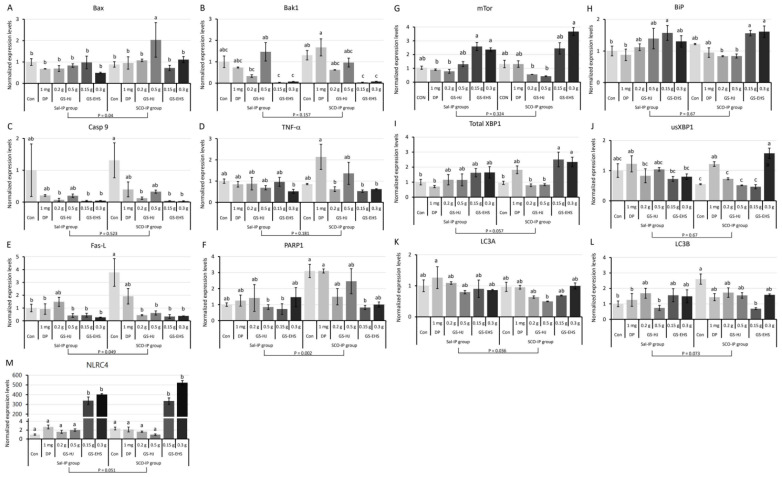
The expression levels of key genes in type I, type II, and inflammatory PCD were altered by GS-EHS supplementation. (**A**). No significant effect of supplementation (*p* = 0.234) or interaction (F_(5, 24)_ = 0.319, *p* = 0.153), but significant effects of IP treatment (*p* = 0.039) in Bax expression. (**B**). Significant effects of supplementation (*p* = 1.0 × 10^−7^) and interaction (F_(5, 24)_ = 2.652, *p* = 0.048), but no effect of IP treatment (*p* = 0.157) in Bak1 expression. (**C**). Significant effect of the supplementation (*p* = 0.007), but no effect of IP treatment (*p* = 0.523) or interaction (F_(5, 24)_ = 0.091, *p* = 0.993) in Casp9 expression. (**D**). Significant effects of supplementation (*p* = 0.029) and interaction (F_(5, 24)_ = 5.018, *p* = 0.003), but no effect of IP treatment (*p* = 0.181) in TNF-α expression. (**E**). Significant effects of supplementation (*p* = 0.0002), IP treatment (*p* = 0.049), and interaction (F_(5, 24)_ = 5.018, *p* = 0.003) in Fas-L expression. (**F**). Significant effects of supplementation (*p* = 0.043), IP treatment (*p* = 0.002), and interaction (F_(5, 24)_ = 3.002, *p* = 0.030) in PARP1 expression. (**G**). Significant effects of supplementation (*p* = 6.1 × 10^−11^) and interaction (F_(5, 24)_ = 5.587, *p* = 0.001), but no effect of IP treatment (*p* = 0.324) in mTor expression. (**H**). Significant effects of supplementation (*p* = 0.003), but no effect of IP treatment (*p* = 0.67) or interaction (F_(5, 24)_ = 1.914, *p* = 0.129) on Bip expression. (**I**). Significant effects of supplementation (*p* = 0.0005), but no effect of IP treatment (*p* = 0.057) or interaction (F_(5, 24)_ = 2.603, *p* = 0.051) in total XBP1 expression. (**J**). Significant effects of supplementation (*p* = 0.0006) and interaction (F_(5, 24)_ = 5.611, *p* = 0.001), but no effect of IP treatment (*p* = 0.254) in usXBP1 expression levels. (**K**). Significant effects of IP treatment (*p* = 0.036), but no effect of supplementation (*p* = 0.092) or interaction (F_(5, 24)_ = 0.965, *p* = 0.458) on LC3A expression. (**L**). No significant effect of supplementation (*p* = 0.115) or IP treatment (*p* = 0.073), but a significant interaction (F_(5, 24)_ = 4.208, *p* = 0.007) on LC3B expression. (**M**). Significant effects of the supplementation (*p* = 2.3 × 10^−21^) and interaction (F_(5, 24)_ = 4.65, *p* = 0.004), but no effect of IP treatment (*p* = 0.051) on NLRC4 expression. Different letters above the bars indicate significant differences (*p* < 0.05). Bak1: BCL2 antagonist killer 1, Bax: BCL2-associated X protein, Casp9: Caspase 9, Con: the control subgroup, DP: donepezil subgroup, Fas-L: fatty acid synthetase-ligand, GS: GS subgroup, GS-EHS: GS enzyme hydrolysate group, LC3A: microtubule-associated protein 1 light chain 3 α, LC3B: microtubule-associated protein 1 light chain 3 β, mTor: mammalian target of rapamycin, NLRC4: NLR family caspase recruitment domain containing protein 4, PARP1: Poly (ADP-ribose) polymerase 1, PCD: programmed cell death, Sal-IP, intraperitoneal injection of saline, SCO-IP: intraperitoneal injection of scopolamine, TNF-α: tumor necrosis factor-alpha, XBP1: X-box-binding protein 1, and usXBP1: un-spliced XBP1.

To investigate whether GS-EHS–induced memory enhancement involves type I PCD, we analyzed the expression of key intrinsic and extrinsic apoptosis regulators and executioners. No significant effect of supplementation (*p* = 0.234) or interaction (F_(5, 24)_ = 0.319, *p* = 0.153)was observed, but a significant effect of IP treatment (*p* = 0.039) was observed in the expression levels of BCL2-associated X protein (Bax), one of the intrinsic apoptosis regulators. Bax expression was highest in the 0.5 g GS subgroup in the SCO-IP group, while 0.3 g GS-EHS subgroup in the Sal-IP group and 0.15 g GS-EHS subgroup in the SCO-IP group showed lower levels than controls (Figure 6A). When the expression levels of BCL2 antagonist killer 1 (Bak1), another intrinsic apoptosis regulator, were analyzed, significant effects of supplementation (*p* = 1.0 × 10^−7^), but not of IP treatment (*p* = 0.157), and a significant interaction was observed (F_(5, 24)_ = 2.652, *p* = 0.048). Bak1 expression was significantly lower in the 0.15 g and 0.3 g GS-EHS under both groups (Figure 6B). When the expression levels of Caspase 9 (Casp9), the other intrinsic apoptosis regulator, were analyzed, significant differences among supplemented groups (*p* = 0.007), but no effect of IP (*p* = 0.523) or interaction (F_(5, 24)_ = 0.091, *p* = 0.993) were observed. Casp9 levels were highest in the SCO-IP Con subgroup, but significantly reduced in the 0.2 g GS, 0.15 g GS-EHS, and 0.3 g GS-EHS (Figure 6C).

When the expression levels of tumor necrosis factor-alpha (TNF-α), one of the extrinsic apoptosis regulators, were analyzed, significant effects of supplementation (*p* = 0.029) and interaction (F_(5, 24)_ = 5.018, *p* = 0.003), but not of IP treatment (*p* = 0.181), were observed. TNF-α expression was elevated in the DP in the SCO-IP group but reduced in the 0.3 g GS-EHS in the Sal-IP group, and in the 0.2 g GS, 0.15 g GS-EHS, and 0.3 g GS-EHS in the SCO-IP group (Figure 6D). The results of the analysis of expression levels of fatty acid synthetase-ligand (Fas-L), another extrinsic apoptosis regulator, showed significant effects of supplementation (*p* = 0.0002), IP treatment (*p* = 0.049), and their interaction (F_(5, 24)_ = 5.018, *p* = 0.003). Fas-L levels were highest in the Con in the SCO-IP group, and significantly lower in the 0.15 g and 0.3 g GS-EHS in both IP groups, with levels less than half those of the Con in the Sal-IP group (Figure 6E). The expression levels of Poly (ADP-ribose) polymerase (PARP1), one of the apoptotic executioners, showed significant effects of the supplementation (*p* = 0.043), IP treatment (*p* = 0.002), and interaction (F_(5, 24)_ = 3.002, *p* = 0.030). PARP1 expression in the Con and DP in the SCO-IP group were significantly higher than those in the 0.5 g GS and 0.15 g GS-EHS in the Sal-IP group and the 0.15 g GS-EHS in the SCO-IP group (Figure 6F). No significant effects of the supplementation (*p* = 0.775), IP treatment (*p* = 0.313), or interaction in the expression of Cytochrome C (CytC) (F_(5, 24)_ = 1.382, *p* = 0.266) (Supplemented Figure S4A) were observed.

#### 3.5.2. Type II PCD-Associated Gene Expression Changes (mTOR, Bip, XBP1, sXBP1, usXBP1, LC3A, LC3B)

Since autophagy/UPR are key players in type II PCD, the expression levels of genes in autophagy/UPR were investigated. When the expression levels of the mammalian target of rapamycin (mTOR), a key regulator of autophagy/UPR [19], was analyzed, significant effects of supplementation (*p* = 6.1 × 10^−11^) and interaction (F_(5, 24)_ = 5.587, *p* = 0.001), but no effect of IP treatment (*p* = 0.324), were observed. mTOR expression was significantly elevated in the 0.15 g and 0.3 g GS-EHS in the Sal-IP and SCO-IP groups compared to the DP and 0.2 g GS in the Sal-IP group and 0.2 g and 0.5 g GS in the SCO-IP group (Figure 6G). Significant effects of supplementation (*p* = 0.003), but not of IP (*p* = 0.67) or interaction (F_(5, 24)_ = 1.914, *p* = 0.129), were observed on the expression levels of BiP, the endoplasmic reticulum resident molecular chaperone binding protein functioning as an UPR sensor [20]. BiP expression was increased in the 0.15 g GS-EHS in the Sal-IP group and 0.15 g and 0.3 g GS-EHS in the SCO-IP group, compared to the Con and DP in the Sal-IP group, and the 0.2 g and 0.5 g GS in the SCO-IP group (Figure 6H). Significant effects of supplementation (*p* = 0.0005), but no IP treatment (*p* = 0.057) or interaction (F_(5, 24)_ = 2.603, *p* = 0.051), were observed in the expression levels of total X-box-binding protein 1 (XBP1), a key modulator of UPR [21]. Total XBP1 contained spliced and un-spliced (sXBPs and usXBP1). Significant effects of supplementation (*p* = 0.0006) and interaction (F_(5, 24)_ = 5.611, *p* = 0.001), but not of IP (*p* = 0.254), were observed in usXBP1 expression. usXBP1 expression was higher in the DP in the Sal-IP group and DP and 0.3 g GS-EHS in the SCO-IP group than in the Con, 0.2 g GS, 0.5 g GS, and 0.15 g GS-EHS in the SCO-IP group (Figure 6I). Cleavage of usXBP1 by Inositol-requiring transmembrane kinase/endoribonuclease 1α (IRE1α) transforms to functional sXBP1 which regulates the expression of UPR target genes [20]. The sXBP1 expression level analysis results showed no significant effect of supplementation (*p* = 0.545), IP (*p* = 0.782), or interaction (F_(5, 24)_ = 0.921, *p* = 0.484) (Supplemented Figure S4B). When the expression levels of microtubule-associated protein 1 light chain 3 α (LC3A) were analyzed, significant effects of IP treatment (*p* = 0.036), but no effect of supplementation (*p* = 0.092) or interaction (F_(5, 24)_ = 0.965, *p* = 0.458), were observed. The LC3A expression levels was lower in the 0.15 GS-EHS in the SCO-IP group than that in the DP in the Sal-IP group (Figure 6K). The microtubule-associated protein 1 light chain 3 β (LC3B) expression level analysis results showed no significant effect of supplementation (*p* = 0.115) or IP treatment (*p* = 0.073), but significant interaction effects (F_(5, 24)_ = 4.208, *p* = 0.007) were observed. LC3B expression was highest in the Con in the SCO-IP group and significantly reduced in the 0.15 g GS-EHS in the SCO-IP group and several Sal-IP subgroups (Figure 6L). The key genes regulating autophagy/UPR-involved type II PCD, such as mTor, Bip, and XBP1, were significantly up-regulated in the 0.15 g GS-EHS and 0.3 g GS-EHS in the SCO-IP group.

#### 3.5.3. Inflammatory PCD-Associated Gene Expression Changes (IL-10R2, NLRC4, UCP2)

Inflammatory PCD also known as pyroptosis are regulated by activated immune cells [22]. Thus, the expression changes in inflammation-involved and inflammasome-formation genes (Supplementary Table S1) were investigated. Of the genes tested, only Interleukin 10 receptor, beta subunit (IL-10R2), NLR family caspase recruitment domain containing protein 4 (NLRC4), and uncoupling protein 2 (UCP2) mRNAs were detected from the mouse brains. No significant effect of supplementation (*p* = 0.382), IP treatment (*p* = 0.279), or interaction (F_(5, 24)_ = 2.216, *p* = 0.086) on the expression levels of IL-10R2 (Supplementary Figure S4C). The UCP2 expression level analyzing data showed no significant effect of supplementation substances (*p* = 0.308), IP treatment (*p* = 0.954), or interaction (F_(5, 24)_ = 1.331, *p* = 0.285) (Supplementary Figure S4D). However, the NLRC4 expression analyzing results showed significant effects of supplementation (*p* = 2.3 × 10^−21^) and interaction (F_(5, 24)_ = 4.65, *p* = 0.004), but no effect of IP treatment (*p* = 0.051). NLRC4 expression was markedly upregulated in the 0.15 g and 0.3 g GS-EHS compared to all other subgroups in both IP groups (Figure 6M). These results suggested that 0.15 g and 0.3 g GS-EHS might activate NLRC4-dependent inflammasome formation in neurons.

These gene expression level analysis results suggested that GS-EHS supplementation may inhibit the progression of type I, type II, and inflammatory PCD by regulating the expression of key genes in apoptosis, autophagy/UPR, and inflammation. This molecular regulation may underlie the neuroprotective effects of GS-EHS and help explain the improved memory performance observed in the behavioral assays.

## 4. Discussion

In this study, we investigated the memory enhancement effects of GS-EHS and the underlying biochemical, cellular, and molecular biological mechanisms using an MCI mouse model, which represents the pre-dementia stage. The MCI model used in this study is induced by SCO-IP, an antagonist of the muscarinic acetylcholine receptor, and is widely used for the development of memory-enhancing substances and the investigation of signal transduction mechanisms underlying learning and memory [23,24]. Since both mouse and rat SCO-IP MCI models effectively replicate cholinergic-based memory impairments, we selected the mouse model for this study. Furthermore, many previous studies, including ours [10,11], have utilized SCO-IP MCI mouse models to evaluate the memory-enhancing effects of foods or drugs [25]. Thus, the use of a mouse model was appropriate for assessing the efficacy of GS-EHS in improving memory function.

GS-EHS was selected because it is derived from HongJam, a natural food sold in Korea [5], which consists of approximately 70% protein by dry weight and contains various phytochemicals originating from mulberry leaves [26]. Much of the protein in HongJam originates from the large quantities of silk protein stored in the enlarged silk glands of mature silkworms. These silk glands also contain functional phytochemicals bound to silk proteins that contribute to the color of the silk fiber [27]. The main components of these fibers are high-molecular-weight fibroin macromolecules [7,28]. Fibroin contains repeated GAGAG(X, XY, or XYZ) amino acid sequences. The silk proteins exist in a soluble form in the glands but transform into highly stable β-sheet-rich fibers upon spinning [29,30]. Because of this stable fiber structure, silk proteins are difficult to digest and absorb when consumed. However, previous studies have shown that enzymatic hydrolysis of fibroin extracted from cocoons into low-molecular-weight peptides improves memory in both animals and humans [8,31], and fibroin hydrolysate is currently sold as a health functional food for memory improvement in Korea [5]. Our previous research also showed that HongJam consumption enhances memory [10,11]. Therefore, we hypothesized that digesting HongJam with food-grade enzymes would increase the digestibility and absorption of silk proteins, potentially resulting in greater memory enhancement compared to HongJam itself. Supporting this, we previously demonstrated that HongJam hydrolyzed with food-grade protease improved Parkinson’s disease onset prevention in a *Drosophila* model [32]. In the present study, GS-EHS processed with another protease significantly enhanced fear-aggravated memory, linked to hippocampal function (Figure 2). Interestingly, memory enhancement effects with 0.15 g and 0.3 g GS-EHS were similar and comparable to 0.5 g GS, supporting that enzymatic digestion generates low molecular weight peptides that are more easily absorbed. Furthermore, antioxidant phytochemicals tightly bound to silk fibers or cells may be released and absorbed more efficiently, allowing lower doses of HongJam to achieve comparable cognitive benefits. These findings suggest that the dose optimization of GS-EHS could enhance both the cost-effectiveness and safety profile of this intervention, making it a promising candidate for long-term use in memory support. Additionally, we confirmed that the GY dipeptide serves as a marker compound for GS-EHS (Supplementary Figure S1), corroborating these findings.

For a compound to exert memory-enhancing effect, it must cross the blood–brain barrier (BBB) and activate various cellular functions within the brain. A variety of bioactive peptides with memory-improving effects have been identified [33]. Enzymatic hydrolysates derived from shrimp [34], soybean [35], Lantern fish [36], and sea cucumber [37] have been reported to exhibit in vitro antioxidant and neuroprotective effects, as well as in vivo learning and memory-promoting effects. Low-molecular-weight peptides (2–20 amino acids) have been shown to cross the BBB via passive diffusion, carrier-mediated transport, receptor-mediated transcytosis, and adsorption-mediated transcytosis [33,38]. Although specific silk protein-derived peptides with memory-enhancing effects have not been clearly identified, it is presumed that the abundant low-molecular-weight peptides in GS-EHS—such as GY dipeptides—cross the BBB and act on brain cells similarly to other memory-enhancing peptides.

SCO-IP disrupts memory acquisition and consolidation by impairing signaling mechanisms that regulate synaptic plasticity [24,39]. In SCO-IP-induced MCI mouse brains supplemented with GS-EHS, we observed increased levels of BDNF, total CREB1, and phosphorylated CREB (p-CREB), consistent with effects reported for other memory-enhancing substances [25,40,41] (Figure 4). Notably, peptides with memory-enhancing effects have been shown to activate the BDNF-CREB signaling pathway [33,34,35,37]. Therefore, low molecular weight peptides in GS-EHS may activate this pathway, enhancing synaptic plasticity and promoting memory improvement.

SCO-IP also induces oxidative stress and genomic DNA damage in brain tissue, leading to mitochondrial dysfunction, which further increases PCD and inflammatory responses [25,39]. Mitochondria are the central hub of cellular metabolism, regulating cellular energy supply, PCD, anti-oxidative stress, ROS generation, fatty acid synthesis, oxidative phosphorylation, and thermogenesis [42]. SCO-IP induced results in the cellular and tissue-level dysfunctions described above. Our previous research demonstrated that HongJam supplementation restored SCO-IP induced reductions in MitoCom I–IV activities and ATP levels [10,11]. In the current study, GS-EHS treatment showed a similar restorative effect, with MitoCom I-IV increasing from 60–70% of normal levels in the Sal-IP group. GS-EHS also improved ATP production more effectively than 0.5 g GS (Figure 3). Additionally, it significantly reduced ROS levels and increased total esterase activity, enhancing detoxification capacity (Figure 5).

Given mitochondria’s crucial role in regulating PCD [42,43], we compared gene expression related to type I, type II, and inflammatory PCD (Figure 6). SCO-IP increased the expression of extrinsic/intrinsic apoptosis regulators and apoptosis executioners that activate type I PCD; GS-EHS supplementation significantly decreased or tended to decrease their expression (Figure 6A–F). Genomic DNA damage, common in various degenerative diseases such as dementia, cancers, and premature aging, is regulated by the DNA damage response (DDR) network [44]. A key nuclear protein in DDR is PARP (Figure 6F), with PARP1 playing vital roles in DNA repair, ribosome biogenesis, and transcriptional regulation [45]. Notably, elevated PARP1 expression in the SCO-IP group was reduced to levels comparable to the Con in the Sal-IP group upon GS-EHS supplementation, suggesting GS-EHS may suppress genomic DNA break formation.

GS-EHS also increased the expression of genes regulating type II autophagy/UPR PCD (Figure 6G–L). Furthermore, the expression of NLRC4, part of the inflammasome complex that regulates inflammation and innate immunity [46], was markedly elevated by GS-EHS (Figure 6M). These results indicate that GS-EHS supplementation inhibits SCO-IP-induced apoptosis, activates autophagy/UPR to alleviate ER stress, and enhances NLRC4 inflammasome formation—collectively contributing to improved memory. While the NLRC4 inflammasome is primarily known for its role in immune defense against bacterial infections [46,47], its elevated expression in this context suggests a potential role in modulating neuroinflammatory responses relevant to cognitive function. One possibility is that NLRC4 activation may influence cytokine release, such as IL-1β or IL-18 [48,49], which can regulate synaptic plasticity and neurogenesis. Additionally, NLRC4 may affect microglial activation states [50], shifting them toward a neuroprotective phenotype that supports memory consolidation. These speculative pathways warrant further investigation to clarify the precise role of NLRC4 in memory enhancement.

This study has several limitations that should be acknowledged. First, while the findings demonstrate promising effects of GS-EHS in a murine model, the lack of human clinical data limits the generalizability of the results. Second, natural extracts such as GS-EHS may have batch-to-batch variability in composition, which could affect reproducibility and consistency in future applications. Third, this study used only male mice, which may not fully capture sex-specific responses to the treatment. Finally, the SCO-induced MCI model represents an acute form of cognitive impairment, whereas many neurodegenerative conditions in humans are chronic and progressive. Future studies using chronic models and including both sexes, as well as clinical validation, are necessary to fully evaluate the therapeutic potential of GS-EHS.

## 5. Conclusions

This study demonstrated that GS-EHS supplementation effectively restored or enhanced various signaling mechanisms impaired by SCO-IP-induced memory loss and suppressed neuronal death mediated by various forms of PCD. Our results highlight improved mitochondrial function as the central mechanism underlying these neuroprotective and memory-enhancing effects. Future research should aim to identify the specific bioactive compounds within GS-EHS capable of crossing the BBB and directly enhancing mitochondrial function in the brain. However, this study is limited by the use of a single preclinical animal model, and the relevance of these findings to human physiology remains to be confirmed. Therefore, future clinical studies will be essential to validate the translational potential of GS-EHS in human populations.

## Data Availability

The authors declare that data supporting the findings of this study are available within this paper.

## Supplementary Materials

The following supporting information can be downloaded at https://www.mdpi.com/article/10.3390/nu17122044/s1, Table S1: The list of genes and nucleotide sequences of oligomers used for RT-q-PCR analyses; Figure S1: UHPLC-MS/MS total ion chromatogram (TIC) and mass spectra of the standard solution of glycine–tyrosine dipeptide and the test solution of GS-EHS; Figure S2: Comparison of spatial memories among experimental animals supplemented with DP, GS, or GS-EHS; Figure S3: Comparison of novel objective assay results among tested mice supplemented with DP, GS, or GS-EHS; and Figure S4: The expression levels of CytC, sXBP1, IL-10R2, and UCP2 were not altered by supplementing GS-EHS.

## Abbreviations

The following abbreviations are used in this manuscript:

A600nm	The absorbance at 600 nm
AD	Alzheimer’s disease
Bak1	BCL2 antagonist killer 1
Bax	BCL2-associated X protein
BDNF	Brain-derived neurotrophic factor
BW	Body weight
Casp 9	Caspase 9
CREB1	Cyclic AMP response element binding protein 1
DCIP	Dichlorophenolindophenol
DP	Donepezil
DTT	Dithiothreitol
Fas-L	Fatty acid synthetase-ligand
GS	Golden Silk HongJam
GS-EHS	Enzyme hydrolysates of GS
GY	Glycine–tyrosine
IL-10R2	Interleukin 10 receptor, beta subunit
LC3A	Microtubule-associated protein 1 light chain 3 α
LC3B	Microtubule-associated protein 1 light chain 3 β
MCI	Mild cognitive impairment
MEB	Mitochondria extraction buffer
MitoCom	Mitochondria complex
mTOR	Mammalian target of rapamycin
NLRC4	NLR family caspase recruitment domain containing protein 4
NOR	Novel objective recognition assay
PAT	Passive avoidance test
PBT	0.1 M phosphate buffer with 1.0% Triton X-100
PCD	Programmed cell death
ROS	Reactive oxygen species
Sal-IP	Intraperitoneal injection of saline
SAR	Spontaneous alteration rate
SCO-IP	Intraperitoneal injection of scopolamine
sXBP1	Spliced XBP1
TNF-α	Tumor necrosis factor alpha
UCP2	Uncoupling Protein 2
UHPLC-MS/MS	Ultra-high performance liquid chromatography-tandem mass spectrometry
UPR	Unfolded protein response
usXBP1	Un-spliced XBP1
XBP1	X-box-binding protein 1
