# Supplementary material for: Silkworm Enzyme Hydrolysates Improve Memory in MCI Models via CREB-BDNF Signaling and Enhanced Brain Mitochondrial Function
Source: Nutrients. 2025 Jun 19;17(12):2044. doi: 10.3390/nu17122044 (PMC12196421; doi:10.3390/nu17122044)
Supplement: Supplementary file 1 [file nutrients-17-02044-s001.zip › nutrients-3692845-supplementary.pdf]

Table S1. The list of genes and nucleotide sequences of oligomers used for RT-q-PCR analyses

Signal transduction pathway		Genes		Nucleotide sequences	RT-q-PCR thermal profile
Type I Programmed Apoptotic cell death	Intrinsic Apoptosis regulators	Apaf-1	F	AGT AAT GGG TCC TAA GCA TGT TG	
			R	GCG ATT GGG AAA ATC ACG TAA AA	
		Bcl2	F	GTG GTG GAG GAA CTC TTC AG	1 x 95°C, 3 min; 40 x (95°C, 5 sec + 60°C, 10 sec + 72 °C, 10 sec)
			R	GTT CCA CAA AGG CAT CCC AG	
		Bax	F	AGC AAA CTG GTG CTC AAG GC	
			R	CCA CAA AGA TGG TCA CTG TC	
		Bak1	F	CAA CCC CGA GAT GGA CAA CTT	
			R	CGT AGC GCC GGT TAA TAT CAT	
	Extrinsic apoptosis regulators	Casp 9	F	AGT TCC CGG GTG CTG TCT AT	
			R	GCC ATG GTC TTT CTG CTC AC	
		TNF-α	F	CCC TCA CAC TCA GAT CAT CTT CT	
			R	GCT ACG ACG TGG GCT ACA G	
		Fas-L	F	GCA GCC CTT CAA TTA CCC AT	
			R	CAG AGG TTG GAC AGG GAA GAA	
		Fas		TGA AGG ACA TGG CTT AGA AGT G	
				GGT GCA AGG GTC ACA GTG TT	
	Apo	PARP1	F	GGC AGC CTG ATG TTG AGG T	

			R	GCG TAC TCC GCT AAA AAG TCA C	
		Casp 3		CCT CAG AGA GAC ATT CAT GG	
				GCA GTA GTC GCC TCT GAA GA	
		Cyt C	F	AGT TCC CGG GTG CTG TCT AT	
			R	TAC TCC ATC AGG GTA TCC TC	
Type II Programmed non-Apoptotic cell death	Autophagy and Unfolded protein response	mTor	F	TCC TGC GCA AGA TGC TCA TC	1 x 95°C, 3 min; 40 x (95°C, 5 sec + 60°C, 10 sec + 72 °C, 10 sec)
			R	TGT GCT CCA GCT CTG TCA GGA	
		BiP	F	TTC AGC CAA TTA TCA GCA AAC TCT	
			R	TTT TCT GAT GTA TCC TCT TCA CCA GT	
		total Xbp1	F	TGG CCG GGT CTG CTG AGT CCG	
			R	GTC CAT GGG AAG ATG TTC TGG	
		usXbp1	F	CAG CAC TCA GAC TAT GTG CA	
			R	GTC CAT GGG AAG ATG TTC TGG	
		sXbp1	F	CTG AGT CCG AAT CAG GTG CAG	
			R	GTC CAT GGG AAG ATG TTC TGG	
		LC3A	F	TGG TCA AGA TCA TCC GGC	
			R	CTC ACC ATG CTG TGC TGG	
		LC3B	F	TTC TTC CTC CTG GTG AAT GG	
			R	GTG GGT GCC TAC GTT CTC AT	
Inflammatory	Inflammation	IL-1β	F	GAA ATG CCA CCT TTT GAC AGT G	
			R	TGG ATG CTC TCA TCA GGA CAG	

		IL-6	F	CTG CAA GAG ACT TCC ATC CAG	
			R	AGT GGT ATA GAC AGG TCT GTTGG	
		IL-10R2	F	TTT GTC GTG CTG TGG CTC AT	
			R	AGG GAA GGA GAA CAG CAG AA	
		iNOS	F	ACA TCG ACC CGT CCA CAG TAT	
			R	CAG AGG GGT AGG CTT GTC TC	
		NLRP1	F	CAC TGC CCA AGA TTG CTA CA	1 x 95°C, 3 min; 40 x (95°C, 5 sec + 60°C, 10 sec + 72 °C, 10 sec)
			R	CTT CAC TCA GCA CCA GAC CA	
		NLRP3	F	GTG GTG ACC CTC TGT GAG GT	
			R	TCT TCC TGG AGC GCT TCT AA	
		NLRC4	F	CTA CAT TGA TGC TGC CTT GG	
			R	ATC CGT CAC TGC TCA CAC AG	
		UCP2	F	GCT GGT GGT GGT CGG AGA T	
			R	TGA AGT GGC AAG GGA GGT	
		AIM2	F	CAC CCT CAT GGA CCT ACA CTA	
			R	CGT TGT TAG TAA ATC AGC AGT TCT	
Loading control		β-actin	F	GGG ATG TTT GCT CCA ACC AA	
			R	GCG CTT TTG ACT CAG GAT TTA	

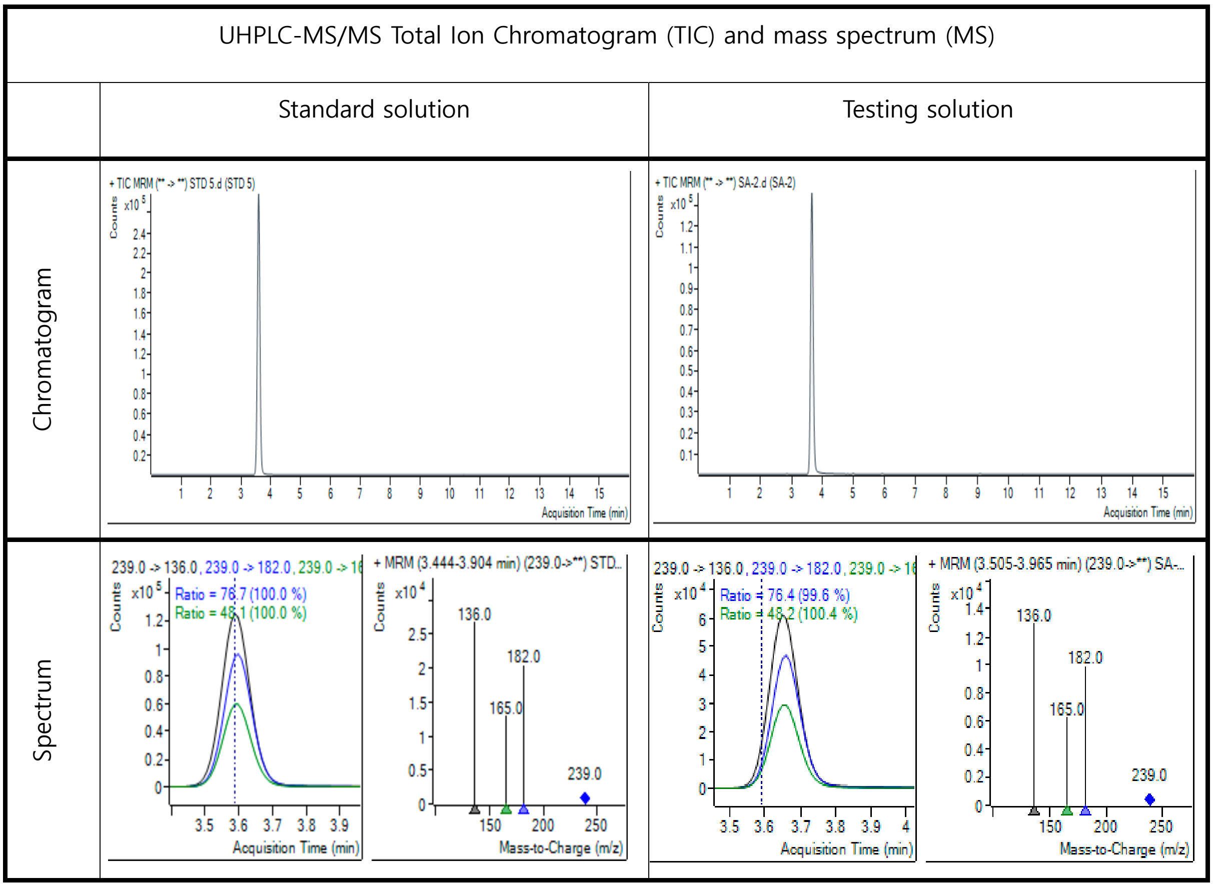

Figure S1. UHPLC-MS/MS total ion chromatogram (TIC) and mass spectra of the standard solution of glycine-tyrosine dipeptide and the test solution of GS-EHS. The TICs and MS spectra of both the standard and test solutions were identical, indicating that glycine-tyrosine dipeptide can serve as a reliable marker compound for GS-EHS.

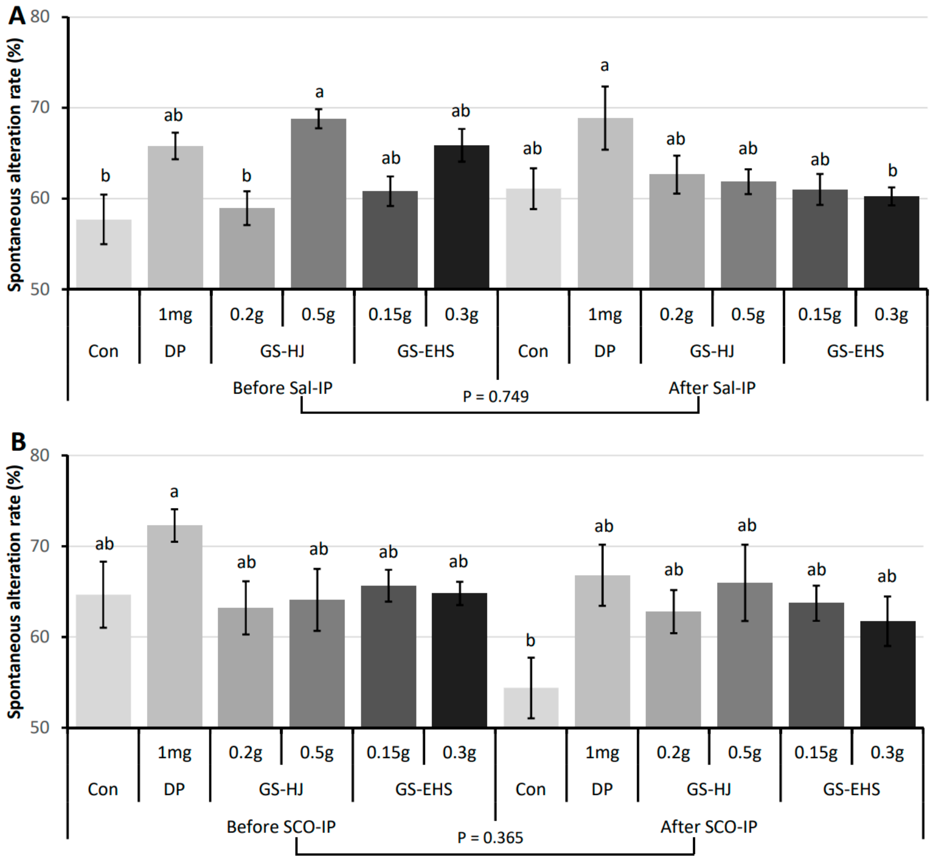

Figure S2. Comparison of spatial memories among experimental animals supplemented with DP, GS or GS-EHS.

A. There were supplemented substance (between) effects (P = 0.000825), but no before-after Sal-IP (within) effects (P = 0.749) in the Sal-IP group. There was no significant within-between interaction effects ($F_{(5, 84)}$ = 2.873, P = 0.019). B. There were between effects (P = 0.03), but no before-after SCO-IP (within) effects (P = 0.055). There was no significant within-between interaction effects ($F_{(5, 84)}$ = 1.101, P = 0.365).

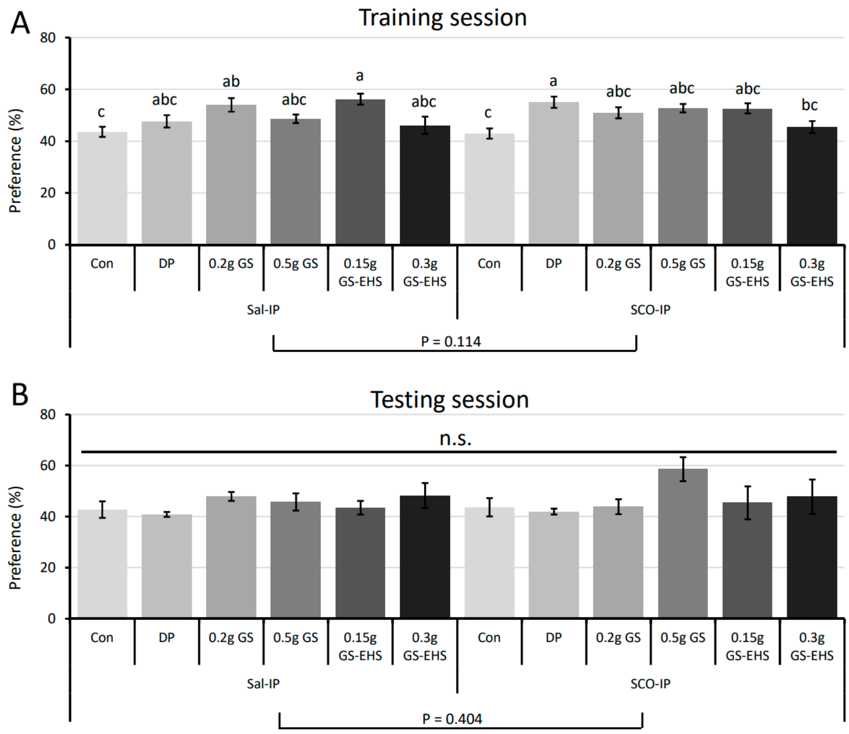

Figure S3. Comparison of novel objective assay results among tested mice supplemented with DP, GS or GS-EHS.

A. There were supplemented substance (between) effects ($P = 1.09 \times 10^{-5}$), but no Sal- or SCO-IP (within) effects (P = 0.65) in the training session. There was no significant within-between interaction effects ($F_{(5, 84)} = 1.836$, P = 0.114). B. There was no between effects (P = 0.109), nor no within effects (P = 0.373) in the testing session. There was no significant within-between interaction effects ($F_{(5, 84)}$ = 1.033, P = 0.404).

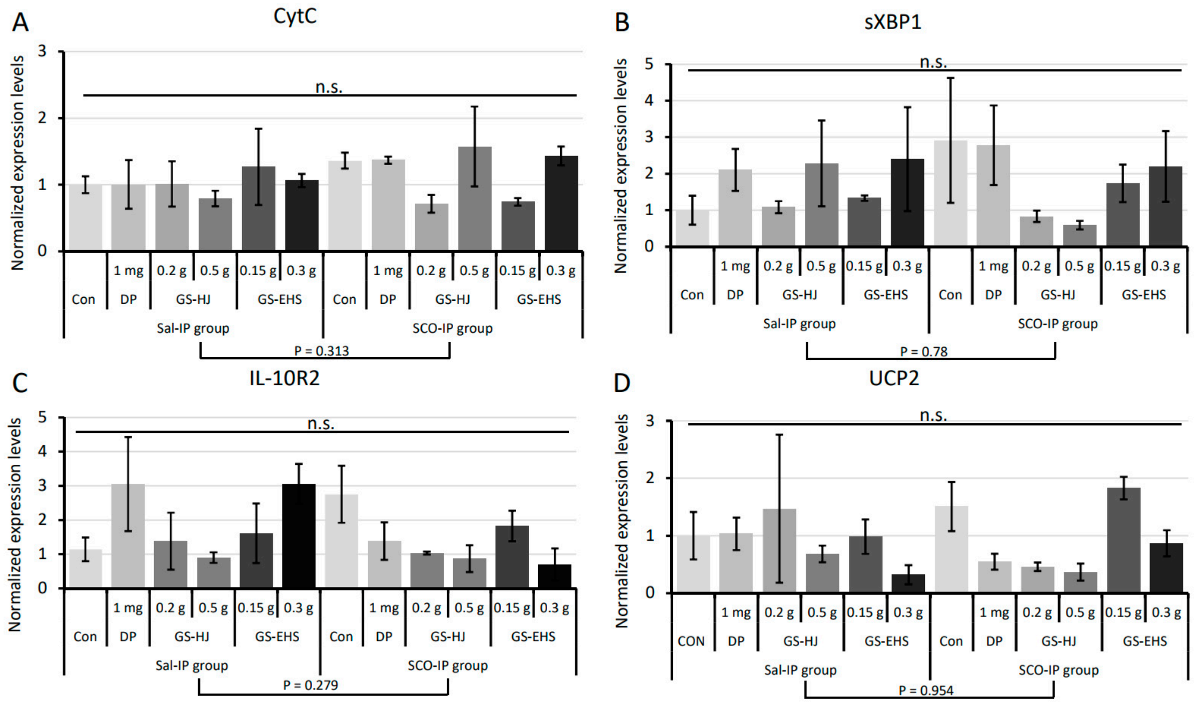

Figure S4. The expression levels of CytC, sXBP1, IL-10R2, and UCP2 were not altered by supplementing GS-EHS. A. There was no significant effect of the supplemented substances (P = 0.775) nor IPs (P = 0.313) in the expression of CytC. There was no interaction effect between the supplemented substances and IPs ($F_{(5, 24)}$ = 1.382, P = 0.266). B. There was no significant effect of the supplemented substances (P = 0.545) nor IPs (P = 0.782) in the expression of sXBP1. There was no interaction between the supplemented substances and IPs ($F_{(5, 24)}$ = 0.921, P = 0.484). C. There was no significant effect of the supplemented substances (P = 0.382) nor IPs (P = 0.279) on the expression levels of IL-10R2. There was no interaction effect between the supplemented substances and IPs ($F_{(5, 24)}$ = 2.216, P = 0.086). D. There was no significant effect of the supplemented substances (P = 0.308) nor IPs (P = 0.954) on the expression of UCP2. There was no interaction between supplemented substances and IPs (F(5, 24) = 1.331, P = 0.285)
